# Effects of Operational Parameters on the Characteristics of Ripples in Double-Pulsed GMAW Process

**DOI:** 10.3390/ma12172767

**Published:** 2019-08-28

**Authors:** Ping Yao, Kang Zhou, Heqing Tang

**Affiliations:** 1 College of Electromechanical Engineering, Guangdong Polytechnic Normal University, Guangzhou 510635, China; 2 School of Mechatronical Engineering, Beijing Institute of Technology, Beijing 100081, China

**Keywords:** DP-GMAW process, weld bead, distance of ripple, twin pulse frequency, twin pulse relation

## Abstract

This study focuses on the characteristics of the ripples of the weld bead formed during the double-pulsed gas metal arc welding (DP-GMAW) process. As a special output of the process, ripples include many useful information and can reflect the quality of the welding process. The work analyzed the operational characteristics of the DP-GMAW process based on robot operation which used the latest *twinpulse* XT DP control process, and then selected five key operational parameters, such as average current, welding speed, twin pulse frequency, twin pulse relation, and twin pulse current change in percent, to explore their effects on the formation and characteristics of ripples. A reliable method of measuring the distance of the ripples was used to provide convincing data. According to a series of experimental observations and analyses, the distance of ripples and appearance under different conditions were obtained. Also, curve fitting equations between each operational parameter and corresponding distances of ripples was obtained. To testify the effectiveness of the curve fitting equations, corresponding verifying experiments were conducted, and the results showed that all the errors were below 10%. In addition, the different levels of the operational parameters on the formation and characteristics of ripples were provided. This work can be a reference for the process and quality control improvement for the DP-GMAW process.

## 1. Introduction

Gas metal arc welding (GMAW) is a commonly used welding process which is used to join a wide range of metallic materials in many industrial fields [1]. As an important improvement for the conventional GMAW process, pulsed GMAW(P-GMAW) process, which has various advantages such as high productivity, fine grain size, and process robustness [2], is being increasingly used for joining a large variety of ferrous and nonferrous materials in reality [3]. Using P-GMAW process technique, a stable droplet transfer can be obtained by detaching the droplet from the wire end at the end of the higher current phase or during the following base current phase [4], and it can produce spray transfer at lower average currents, which can overcome the drawback that good thermal and electrical conductivity during welding process generally results in excessive heating of base material [5]. However, to increase the productivity without quality drops, double-pulsed GMAW (DP-GMAW) process was then used. It was a variation form of P-GMAW process, in which a low frequency current pulse is superimposed into a single high-frequency current pulse [6]. The low-frequency current pulse is called as thermal pulse. Hence, the droplet transfer is simultaneously controlled by the high-frequency pulse and thermal pulse. Compared to the P-GMAW process, the DP-GMAW process can adjust the parameters in a much wider range, so that the process and performance control can be easier. Also, because of the synchronization between welding current and arc voltage during the period of thermal pulse, the deposit of DP-GMAW process introduces comparatively more thermal shock compared to that of P-GMAW process, therefore the heat input was reduced and the properties of the weld joints can be improved [7]. Apart from this merit, it has other advantages than that of conventional P-GMAW process, such as the DP-GMAW process has better gap bridging ability [8], can reduce the porosity content in the weld pool when compared to P-GMAW process and obtain comparatively finer microstructure of weld and heat affected zone which can improve the tensile and hardness properties of the weld joint [9], and can improve the solidification cracking susceptibility [10] and have more wider applying areas than that of P-GMAW process. Hence, this process is a typical low-heat input technique and widely employed in various manufacturing occasions currently, it is being seriously considered by many scholars and experts.

To increase the productivity during the industrial application, the DP-GMAW process was collaborated with robot operation, which can make the operational process convenient and significantly improve the precision of process control, or realize other important functions. Recently, many relative contributions employed robots to improve the arc welding process. Yang et al. [11] employed an arc welding robot to detect the welding quality based on the three-dimensional reconstruction using the shape from shading (SFS) algorithm; the experimental results showed that the system can quickly and efficiently fulfill the detection tasks. Chen et al. [12] used a welding robot to acquire and optimize the weld trajectory and pose information, by means of laser sensor, charge-coupled device (CCD) camera, and other auxiliary instruments. Aviles-Viñas et al. [13] employed an industrial KUKA robot and a GMAW type machine and proposed a real time computer vision algorithm to extract training pattern to acquire knowledge to later predict specific geometries; the final results showed that the accuracy rage can achieve 95%. The arc welding robot was also used in additive manufacturing and has obtained some significant achievements [14]. According to these contributions, it can be noticed that compared to the traditional human-welding, welding process based on robot operation can increase the safety of the operation process, ensure the welding quality, and improve the welding efficiency. Therefore, the arc welding robots can be considered as a typical representative of intelligent manufacturing technology. 

Online evaluation of weld quality is important in all of the welding processes, such as in resistance spot welding, researchers and experts took efforts to improve the tensile–shear strength of the weld [15]. Alternatively, in some metal surface processing area, X-ray and electron microscopy were the main means to detect the formation feature [16]. The output of the DP-GMAW process is the weld bead, hence, the evaluation of the quality of the process is to evaluate the performance of the weld bead. After the parent metal is processed using this technique, the fish scale ripples appear on the bead surface, and the appearance of the ripples can be used to justify whether the welding quality is satisfactory. As an important quality criterion of evaluating the welding quality of the arc welding process, ripples has been seriously considered by many published contributions. Hu et al. [17] used a moving three-dimensional GMAW model to study the transient weld pool dynamic under the periodical impingement of filler droplets that carry mass, momentum, and thermal energy. The work calculated the weld pool shape and distribution of temperature and velocity as a function of time. In addition, according to the analysis of the dynamic mechanics of formation of the ripples, it concluded that the periodic impingement of droplets, weld pool dynamics, and solidification all contributed to the formation of ripples. Moreover, to obtain more detailed results, Rao et al. [18] employed a three-dimensional moving GMAW model to explore the complex transport phenomena and their effects on the formation of the ripples. The effects of some welding process parameters, which included the welding current, drop impinging velocity, and travel speed, on the formation and final appearance were studied for a moving GMAW process. The main calculations included the transient distribution of the melt flow velocity, temperature, and species in the workpiece, as well as the weld pool dynamics and surface rippling on the solidified weld bead. The work revealed the sequence of the ripple formation, and main characteristics of the appearance of the ripple. These two works used the numerical model to explore the effects of various welding parameters on the dynamic process of the weld bead formation. Some important and useful conclusions were drawn, however, the results lacked necessary support from the actual experiments, though there were important references for the experimental operations. Wang et al. [19] researched the effect of thermal frequency, which was the frequency of the low-frequency current pulse for current modulation in DP-GMAW process, on the stability of the welding process, ripple distance, maximum penetration, and grain size and hardness of the fusion zone, though a series of experiments and measurements. The work revealed that the ripple distance reduces as the thermal frequency increases. 

Though there were some relative works which explored the variation characteristics of ripples during the arc welding process, the works were based on the numerical simulation analysis or only few parameters were considered. According to the theoretical analysis, the formation of the weld bead and characteristic of the ripples were related to the multi-parameters. During the DP-GMAW operation process, there are so many process parameters simultaneously influencing the process and output of the system [20], also for the formation and characteristics of the ripples. In this work, some key operational parameters were selected to explore the effects on the formation and characteristics of ripples, and then the rules can also be sought according to the experimental observation and analyses results. 

The rest of the paper is organized as follows. Section 2 provides the key characteristics of DP-GMAW process based on robot operation. In this section some parameters and their meanings during the process are provided. Section 3 details the experimental work, including the experimental platform and main methods. The detailed experiments and corresponding analyses are provided in Section 4. Here, the effects of the five key operational parameters on the formation and characteristic of the ripples are presented, and the curve fitting equations between each parameter and corresponding distances of ripples are also obtained. To obtain effectiveness of the curve fitting equations, Section 5 provides the corresponding testifying experiments. At last Section 6 gives some important conclusions and suggestions for the future works. 

## 2. Key Characteristics of DP-GMAW Process 

**Figure 1:**
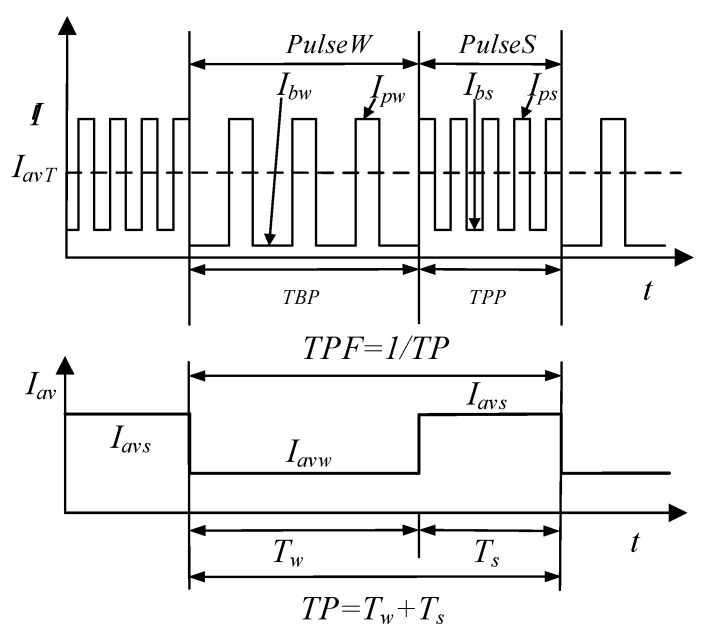
Schematic representation of current pulses during the double-pulsed gas metal arc welding (DP-GMAW) process.

During the DP-GMAW process, the current waveform included two distinct phases, which are thermal pulse phase (TPP) and thermal base phase (TBP). The current pulses in the two phases have different frequencies. The duration of the two phases were respectively marked as *T_s_* and *T_w_*, and one thermal period (TP) was the sum of *T_s_* and *T_w_*. The frequency of the process, which is twin pulse frequency (TPF), is a reciprocal of the TP. TPF is an important parameter which can describe the speed of the current waveform adjustment, and is so related to the formation and characteristics of the ripples. Figure 1 shows the schematic of current pulses during the process.

In Figure 1, there are two high-frequency current pulses existing in the TBP and TPP, the pulses in TPP are stronger than that in TBP. We designated the pulse in TBP as *PulseW*, while the pulse in TPP as *PulseS*. In general case, the frequency of *PulseS* is higher than that of *PulseW*. During the process, TPF can reflect the varying speed of the strong and weak pulses sets, which are respectively *PulseS* and *PulseW*, and each set may include up to 10–20 high frequency pulses, and the maximum frequency may achieve 100 Hz. Hence, in general case, the value of TPF is below 5 Hz. The peak values and base values of the two pulses are named respectively as *I_ps_*, *I_pw_*, *I_bs_*, and *I_bw_*. In addition, the average current in *T_w_* is marked as *I_avw_*, while the average current in *T_s_* is marked as *I_avs_*, and the *I_av_* denotes the average current in one thermal period. Moreover, there are some other important parameters during the process. The first is the twin pulse current change, whose value is half of the difference between average currents in the two phases. This parameter can denote the current variation during two phases. In this work, to clearly reflect the current adjustment during the process, this parameter is described together with the *I_av_* and using percent format, as shown in the following equation:

(1) $$I_{\Delta }=\frac{I_{avs}-I_{avw}}{2I_{av}}\times 100\%$$

where *I_Δ_* denotes the twin pulse current change in percent. In addition, the relations between the average current in two phases and the *I_Δ_* can also be deducted as follows:

(2) $$\left\{ \begin{array}{l} I_{avs}=I_{av}\times (1+I_{\Delta })\\ I_{avw}=I_{av}\times (1-I_{\Delta }) \end{array}\right.$$

Moreover, the proportion of the time of the thermal pulse phase (*T_s_*) in one thermal period (*TP*), is denoted as twin pulse relation in this process, its mathematical description is as follows:

*D_T_* = *T_s_/TP* (3)

where *D_T_* denotes the twin pulse relation in this work. 

According to the above description, there are various operational parameters during the DP-GMAW process, and proper combination of these operational parameters is very important to obtain ripples with satisfactory appearance and quality. However, during the traditional operation process, it is difficult to appropriately adjust all of parameters one by one. In this work, the latest *twinpulse* XT DP control process developed by LORCH Company ( Im Anwänder Auenwald, Germany) was used. The technique used in this equipment has two significant merits. The first is that the current pulse frequencies in TPP and TBP are higher, which can achieve respectively 100 and 30, than those during the traditional DP-GMAW process; while the second is that when the average welding current in one TP can remain constant, and the peak and base values of the current pulses in two phases are unchanged. The adjustment for the pulses in two phases can be conducted by only adjusting the average current *I_av_* and the twin pulse relation *D_T_*. Hence, for double current pulses during the process, this technology can decrease the number of operational parameters which should be adjusted, so that the process control can be more effectively and conveniently executed.

In addition, in this work, an industrial robot was employed to control the operation. In general, the arc welding based on robot operation can accurately control the welding speed in order to assure the same welding time for parent metal with a certain length. Through a special collaborative control instrument, the predetermined operation program can be precisely executed and some important relative process signals can also be collected and analyzed in real time. In addition to accurately control the welding speed, the robot can precisely adjust online the inclination of the electrode. In this work, a fixed inclination of the electrode was used to test the effects of operational parameters on the formation of ripples.

## 3. Experiments of the Work 

### 3.1. Experimental Equipment and Platform

**Figure 2:**
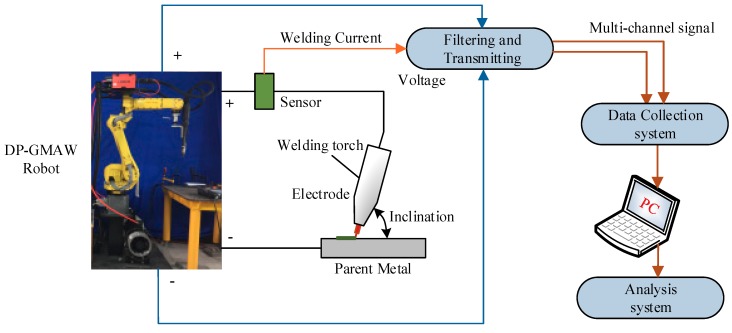
Structure and platform of the work.

To explore the detailed effects of the operational parameters on the formation of the ripples during the DP-GMAW process, corresponding experiments using the DP-GMAW process based on robot operation were conducted in this work. The experiment platform was composed of a FANUS Robot M-10A (FANUS Corporation, Oshino-mura, Yamanashi Prefecture, Japan) industrial robot, a LORCH S-RobotMIG (Lorch Schweißtechnik GmbH, Im Anwänder, Auenwald, Germany) arc welding machine, a wire feeder machine, a welding torch, and some other auxiliary equipment. The industrial robot controlled the welding speed and the inclination of the electrode. During this process, the degree of the inclination of the electrode used the backward inclination mode, which meant that the electrode inclination went along the welding direction. The current waveforms in TPP and TBP were controlled by the LORCH arc welding machine (Lorch Schweißtechnik GmbH, Im Anwänder, Auenwald, Germany). Moreover, a self-designed multi-channel signal collection system and corresponding analysis system were used. Figure 2 shows the experimental structure and platform employed in this work. 

The parent metal in this work used the 304 stainless steel, the tensile strength was 520 MPa, and the size was 250 mm × 60 mm × 3 mm, while the welding wire used the 316 L stainless steel with a diameter of 1.2 mm. Table 1 shows the material characteristics of the parent metal and the welding wire.

In addition, the shielding gas was composed of 98% pure argon and 2% CO_2_ with 15 L/min of velocity. The length of wire extension was 12 mm. The work used the flat surfacing welding. Furthermore, to obtain accurate results to reflect the corresponding effects, the surface of each parent metal should be processed by angle grinder to eliminate the oxides, and then washed by special alcohol. Then, the corresponding welding actions can be started. 

### 3.2. Experimental Method

The DP-GMAW process in this experiment used the *twinpulse* XT DP control process, the changing operational parameters were average current (A), welding speed (cm/min), twin pulse relation (%), twin pulse current change in percent (%), and twin pulse frequency (Hz). To explore the effect of one specific operational parameter on the formation and characteristic of ripples, the values of other operational parameters should be unchanged. Hence, before changing the target operational parameter, one array of reference conditions of operational parameters should be confirmed. 

After some relative trails and analyses, the reference experimental operational parameters are shown in Table 2.

Then to explore the effects of different operational parameters on the variation rules of the characteristic of the ripples, the relative operational parameters were changed when other operational values remained unchanged as in Table 2. 

The experimental process is as follows:

1. The upper and lower limits of each operational parameter were obtained through relative experiments, then for one operational parameter, some testing values following the isometric mode were set, in order to obtain the effect of this operational parameter on appearance and other characteristics of the ripples.
2. Curve fitting tool was used to obtain the equation of experimental results using this operational parameter.
3. Other operational parameters were also employed to use the same method to obtain the corresponding characteristics analyses and equations, and the errors of the curve fitting equations were also obtained.
4. Conclusion of the effect of each operational parameter on the variation rules of the distance of the ripples was drawn.

### 3.3. Measurement Principle and Method

**Figure 3:**
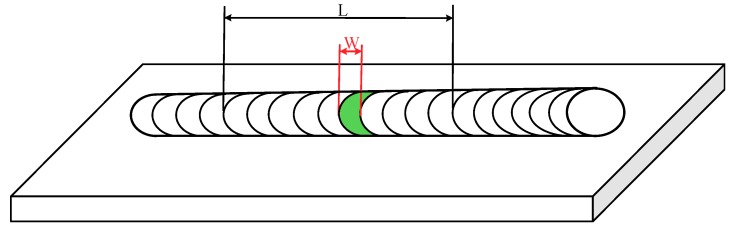
The schematic representation of the distance of ripples and the first measurement method.

In this work, the formation and the characteristics of ripples were seriously considered. For ripples, apart from the appearance observation, other criterion, which is the distance of ripples, can be used to quantitatively describe the characteristic. Hence, this work also employed the distance of ripples as the evaluation criterion for the ripples in each experiment. In general, the distance of ripples referred to the length of the weld bead between the tops of two adjacent ripples, as shown in Figure 3. 

The distance of the ripple is marked as *W* in Figure 3. It can be noticed that *W* is the largest distance between the two adjacent ripples. 

However, during actual welding process, the ripples in one weld bead may be irregular, or the distances were not uniform in reality. Direct measurement of the distance may induce large errors. To decrease the errors and obtain more accurate and reliable results, two measurement methods were designed in this work. 

The first method is shown in Figure 3. Ten ripples were selected in the middle of the weld bead, then the total length can be measured, which was marked as *L*. Hence, the average distance of ripples can be obtained as follows:

*W_av1_* = *L*/10 (4)

**Figure 4:**
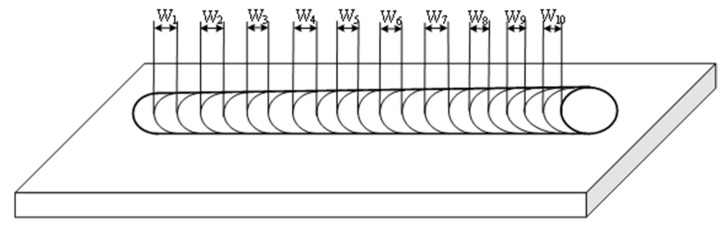
The schematic representation of the second measurement method of the distance of the ripples.

The second measurement is shown in Figure 4. In this method, 10 single ripples were selected and then the distance of each ripple was obtained; then the average distance of the ripples was obtained as follows:

*W_av2_* = (*W_1_* + *W_2_* +…+ *W_9_* + *W_10_*)/10 (5)

In this work, the accuracy of the measurement of the distance of the ripples was very important. Hence, above two measurements were combined. During the data processing, two staffs, who were marked as *A* and *B*, respectively used the first and second methods to obtain corresponding results. Four measurement results were obtained for each weld bead. Then the final distance was the average value of the four results. The calculation can be shown as follows:

*W_av_* = (*W_av1A_* + *W_av1B_* + *W_av2A_* + *W_av2B_*)/4 (6)

It can be noticed that combining two measurement methods and using the average value as the final distance can decrease the systematic error and human measurement error, and measuring several times can decrease the random error. Hence, the final distance using these measurement methods were enough reliable.

### 3.4. Evaluation Method for the Errors of the Curve Fitting

In this work, the measured distance and the variation of the operational parameters can be curve fitted to explore the corresponding effects. To evaluate the performance of the equation using curve fitting, mean squared error (MSE) was employed. The mathematic description of the MSE is as follows:

(7) $$MSE=\frac{1}{m}{\sum_{i=1}^{m}\left(y_{i}-{\hat{y}}_{i}\right)}^{2}$$

where *m* is the number of the values of distance, *y_i_* is the measurement value, ${\hat{y}}_{i}$ is the value obtained from the curve fitting. It can be noticed that smaller *MSE* means more accuracy of the curve fitting. 

## 4. Experiments of Single Operational Parameter and Analyses 

In this section, the effects of the five main operational parameters on the formation and characteristic of ripples are detailed explored based on the corresponding experimental observation and analyses. The detailed experimental plan, which included the information of different factors and levels, is shown in Table 3.

Each main operational parameter corresponded to a special variation range, which is highlighted in bold and underlined in Table 3. 

### 4.1. The Effects of Average Current

In each experiment, the upper and lower limits of the specific parameter were determined by using the reference experimental condition of operational parameters; the experimental interval of the average welding current was 10 A. According to the experimental results, when the average current was below 60 A, the heat delivery to the parent metals was not enough, suggesting that the bead height of the weld bead was so high that the edge of the bead could not be penetrated; and when the average current was above 110 A, excessive heat delivery broke the parent metal and a hole appeared. Hence, the average current in the experiment was selected between 60 A and 110 A with 10A interval. The experimental results are shown in Table 4; the specimens are labelled as I1 to I6. 

It can be noticed that the experiment of specimen I3 used 80 A as the average current, which was same as the reference experimental condition of the average current. Hence, the data of this specimen can also be used in other parts that can analyze the other effects of other parameters. 

**Figure 5:**
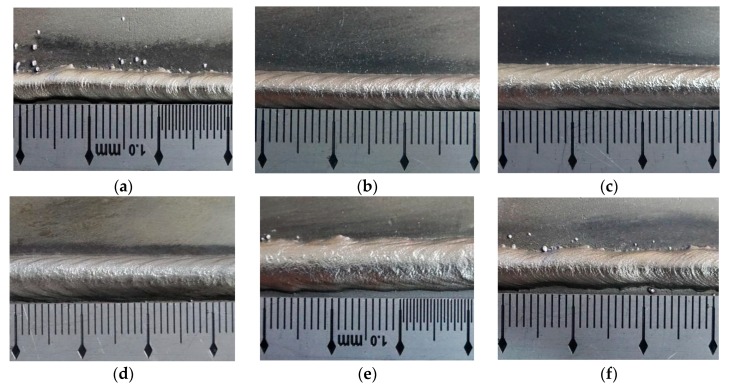
The appearance of the weld bead. (**a**) Specimen I1, (**b**) specimen I2, (**c**) specimen I3, (**d**) specimen I4, (**e**) specimen I5, (**f**) specimen I6.

Corresponding appearances of the ripples are shown in Figure 5. During the experimental process of the specimen of I1, more splashes occurred during the process, and the bead was narrow with large value of bead height. In addition, the ripple was compact, but the bead was not straight, and some unfused parts appeared. In this work, compacted ripples meant that the values of distances of ripples are small. While the weld bead of specimen I2 had a uniform width, stable welding process, and regular ripples, and the distances of the ripples were larger than those of the specimen I1. The weld bead of specimen I3 was so straight with uniform width, and the welding process was stable, as well as the ripples were so clear and had beautiful appearance, also, the bead had silvery appearance and bright luster. The weld bead of specimen I4 was also straight with uniform width, and had a stable welding process, as well as clear and fluent ripples. The bead height of weld bead of specimen I5 was large, and few splashes occurred. The appearance of the bead was dim, and the boundaries of the ripples were not clear. For the weld bead of specimen I6, very large welding current made the formation of the weld bead unsatisfied, where the height and width were not uniform, and the ripples were also irregular and not uniform. According to these six specimens, larger value of average current can induce larger value of the width of the weld beads. Though the distance of ripples grew wider with increasing welding current, the trend was not obvious enough. The distance of the ripples of these specimens were between 2.46 mm and 3.98 mm, and the variation range was only 1.52 mm with the welding current varying from 60 A to 110 A. 

**Figure 6:**
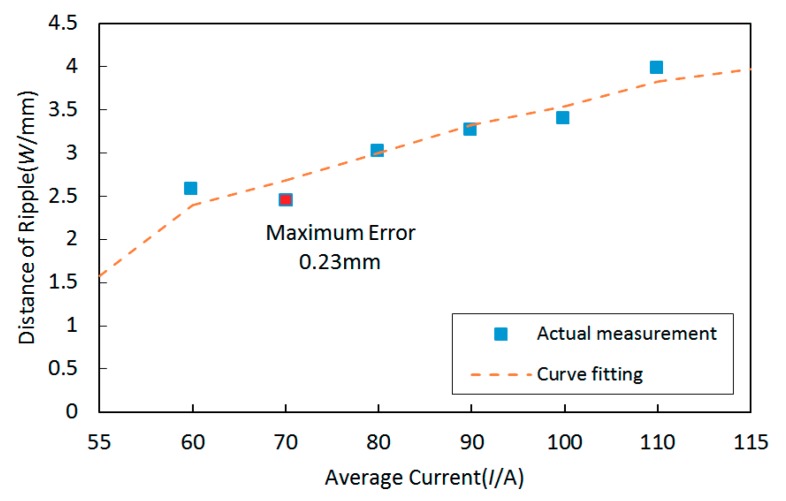
The variation tendency between distances of ripples and the average currents and the curve fitting result.

In addition, the variation tendency between distances of ripples and average currents is shown in Figure 6. It can be noticed that distances of ripples increased as the average current increased, and the variation tendency was approximately linear.

The variation tendency can be curve fitted using the linear equation as follows:

Y = 0.029x + 0.673(8)

The largest curve fitting error was 0.23 mm, and the corresponding MSE was 0.0224. 

### 4.2. The Effects of Welding Speed

To explore the effect of different welding speed on the formation and characteristics of ripples, different welding speeds were employed when other parameters used reference experimental conditions. When the welding speed was below 20 cm/min, the weld bead was so wide and the parent metal was penetrated; with increasing welding speed, the width of weld bead was reducing, when the welding speed achieved 60 cm/min, serious undercut and nodular accumulation appeared, suggesting that the welding speed was so high that the parent metal could not be penetrated. Hence, appropriate welding speed were selected from 20 cm/min to 60 cm/min. The experimental results are shown in Table 5, and the specimens are labelled as S1 to S5. 

**Figure 7:**
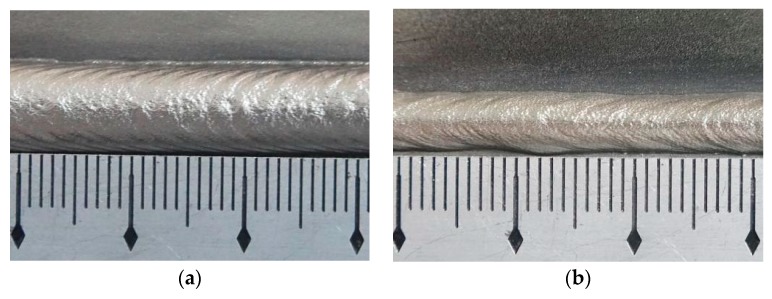

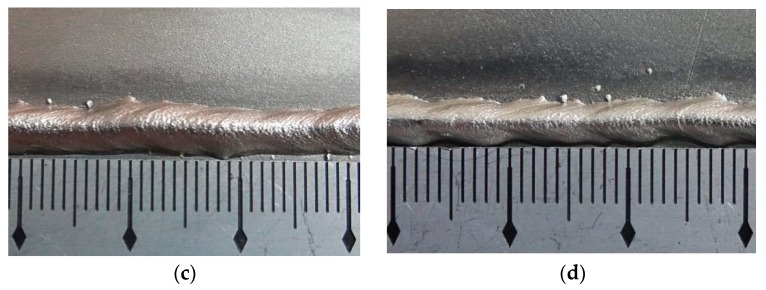
The appearance of the weld bead. (**a**) Specimen S1, (**b**) specimen S3, (**c**) specimen S4, (**d**) specimen S5.

Corresponding appearances of the ripples are shown in Figure 7. The weld bead of specimen S3 was the same as that of specimen I3 in Figure 5c, so only four weld beads are included in Figure 7. The weld bead of specimen S1 had large width and was bright, and the ripple was obvious enough. However, the ripples at the top of the bead were not clear, and some drop-like or lump droplets appeared. The weld bead of specimen S3 had uniform width and the ripples were obvious, only few splashes appeared during the process. On the other hand, the formation of the weld bead of specimen S4 was unsatisfying, the weld bead was narrow and the distances of ripples increased. However, the distances were not uniform, and few splashes appeared. For the weld bead of specimen S5, the width of the weld bead was also small, and the distances of ripples were large. Both the width and the height of the weld bead were not uniform, and nodular accumulation appeared on the bead and more splashes appeared during the process. According to the welding process and formation of ripples, with increasing welding speed, the weld bead turned narrow and the distance of the ripples increased. It may be because one ripple formed during one thermal period under the DP-GMAW process when the average welding and twin pulse frequency were unchanged; larger welding speed meant that the parent metal contacted the welding torch for a longer time during one thermal period, which also increased the distances of the ripples. 

The corresponding variation tendency can be summarized according to Table 3 and Figure 7. It can be noticed that higher welding speed resulted in larger values of the distances of the ripples. The increasing trend was more obvious than that of the average current, and the overall distances were between 1.93 mm and 5.78 mm, the variation range was 3.85 mm. 

**Figure 8:**
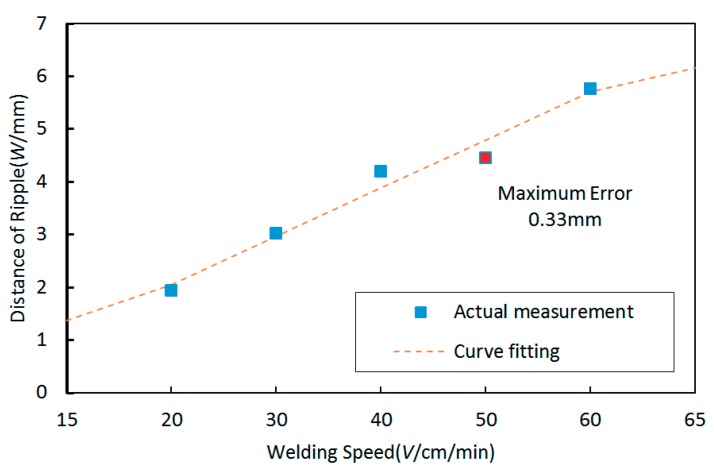
The variation tendency between distances of ripples and welding speed and the curve fitting result.

To obtain more clear variation characteristics, the variation tendency between distances of ripples and welding speed can also be curve fitted as shown in Figure 8. It can be observed that the distances of ripples increased with an increasing welding speed. The variation tendency was still approximately linear. 

The corresponding linear equation from curve fitting is as follows:

y = 0.091x + 0.222(9)

The largest curve fitting error is 0.33 mm, and the corresponding MSE is 0.04684. 

### 4.3. The Effects of Twin Pulse Frequency

In general, the twin pulse frequency of the power source during DP-GMAW process was 0–5 Hz. Different values of twin pulse frequencies were employed in this experiment to determine a proper variation range of the parameter by using the reference experimental condition of operational parameters. Considering when the twin pulse frequency was below 1 Hz, the process was approximately a traditional P-GMAW process and the ripples abnormally appeared. Hence, the twin pulse frequency was selected from 1.5 Hz to 5 Hz, which included 6-array results. The experimental results are shown in Table 6. 

**Figure 9:**
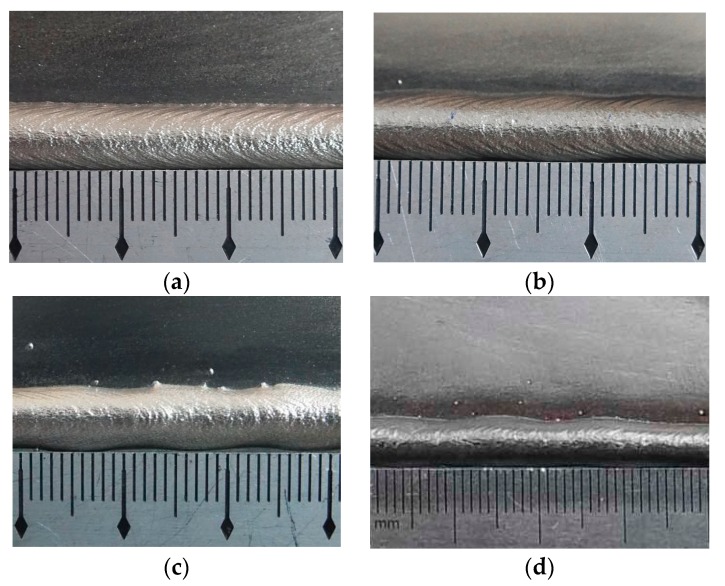
The appearance of the weld bead. (**a**) Specimen F2, (**b**) specimen F3, (**c**) specimen F4, (**d**) specimen F5.

The appearances of the ripples when used different twin pulse frequencies are shown in Figure 9. The weld bead of specimen F1 was the same as that of specimen I3 in Figure 5c, so Figure 9 included four weld beads. The weld bead of specimen F2 had uniform width, and the ripples were so compact and clear, also, the weld bead had good luster. Also, the weld bead of specimen F3 had uniform width and compact ripples. The width of the weld bead of specimen F4 was not uniform; however, the ripples were also compact and clear, and the weld bead also had good luster. The last weld bead, which corresponded the specimen F5, was the most compacted when compared to other ones, and the width was still a little not uniform and so small. It can be noticed that the higher twin pulse frequency can induce more compacted ripples. This showed that an increase in twin pulse frequency resulted in thermal period reduction. Under the situation of unchanged welding current and welding speed, lower thermal period meant that shorter weld bead can be formed during one thermal period. One ripple can be formed within one thermal frequency during the DP-GMAW process. Hence, higher twin pulse frequency corresponds to more compacted ripples, where distance of ripples were smaller, and hence the variation tendency was obvious. During these experiments, the distances of the ripples were between 0.98 mm and 3.02 mm, and the variation range was 2.04 mm. 

**Figure 10:**
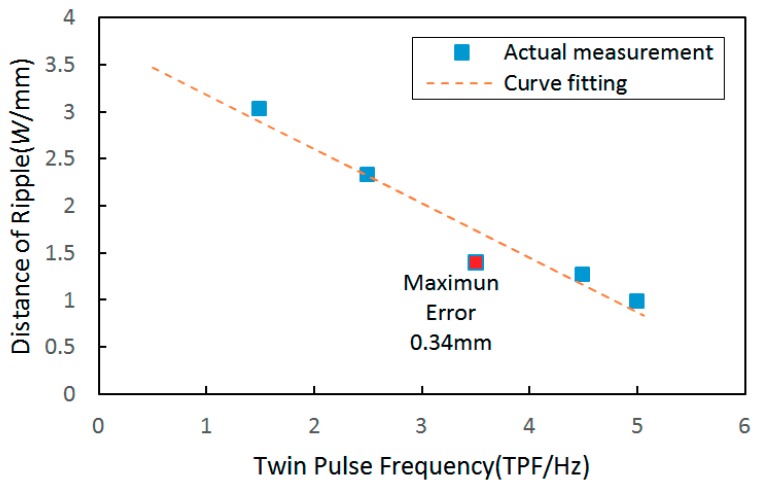
The variation tendency between distances of ripples and twin pulse frequency and the curve fitting result.

The variation tendency between twin pulse frequencies and distances of the ripples is shown in Figure 10. It can be noticed that higher twin pulse frequency induced more compacted ripples, and the corresponding variation tendency was also approximately linear. 

The corresponding curve fitting equation between twin pulse frequencies and distances of the ripples can be described as follows:

y = −0.577x + 3.758(10)

The largest curve fitting error was 0.34 mm, and the corresponding MSE was 0.03092.

### 4.4. The Effects of Twin Pulse Relation

In general, the variation range of the twin pulse relation was from 20% to 80%. Hence, seven values of the twin pulse relations were employed in this experiment, when other parameters used reference experimental conditions. The experimental results are shown in Table 7.

**Figure 11:**
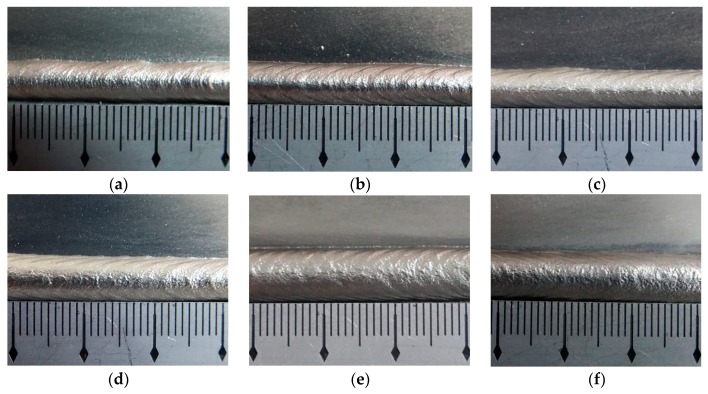
The appearance of the weld bead. (**a**) Specimen R1, (**b**) specimen R2, (**c**) specimen R3, (**d**) specimen R5, (**e**) specimen R6, (**f**) specimen R7.

The appearances of the ripples when employed different twin pulse relations are shown in Figure 11. The weld bead of specimen R4 was the same as that of specimen I3 in Figure 5c, so Figure 11 included six weld beads. All of the weld beads in Figure 11 had uniform widths. The ripples in the weld bead of specimen R1 were not clear. The distance of ripples of the weld bead of the specimen R2 was large. While the ripples in the weld bead of specimen R3 were clear enough. Also, ripples in the weld bead of specimen R4 and R5 were clear. The weld bead of specimen R6 also had compacted and clear ripples. The last was the weld bead of specimen R7, the ripples were more compacted than that of R6; however, the ripples at the top of the weld bead were not clear, it may be due to some blocky fusion zones resulting from high twin pulse relation made the boundaries of the ripples vague. According to these experiments, it can be observed that with the increasing of the twin pulse relation, the width of the weld bead was increasing and the distances of the ripples were decreasing, which can obtain more compacted ripples. This was because that the twin pulse relation can affect the average current, larger value of twin pulse relation meant larger value of average current. The overall distances of the ripples were between 2.73 mm and 3.89 mm, and the variation range was 1.16 mm, which meant that the effect of the twin pulse relation on the distances of ripples was not significant. 

**Figure 12:**
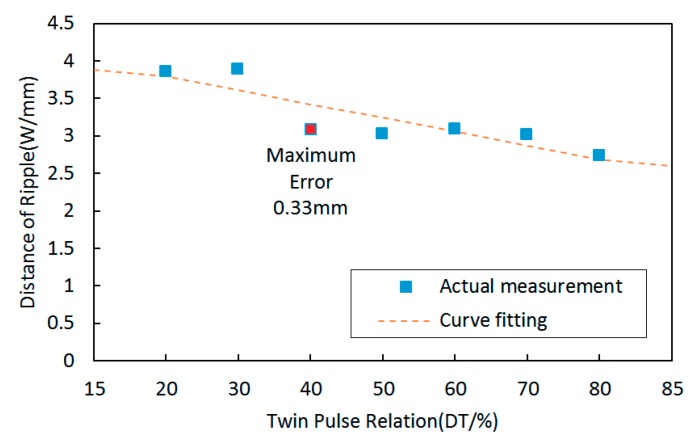
The variation tendency between distances of ripples and twin pulse relation and the curve fitting result.

The variation tendency between twin pulse relations and distances of the ripples is shown in Figure 12. It can be observed that more and more compacted ripples can be obtained with the increasing of the twin pulse relation, and the variation tendency was also approximately linear. 

The corresponding curve fitting equation between twin pulse relations and distances of the ripples can be described as follows:

y = −0.018x + 4.154(11)

The largest curve fitting error was 0.33 mm, and the corresponding MSE was 0.0373. 

### 4.5. The Effects of Twin Pulse Current Change in Percent

In general, the variation range of the twin pulse current change in percent was between 0 and 50%. In this part, the parameters apart from twin pulse current change in percent remained unchanged. Considering when the twin pulse current change in percent was 0, the process was also a traditional P-GMAW process and no ripples appeared. Hence, six values, which were 5%, 15%, 25%, 35%, 45%, and 50% were selected. The experimental results are shown in Table 8.

**Figure 13:**
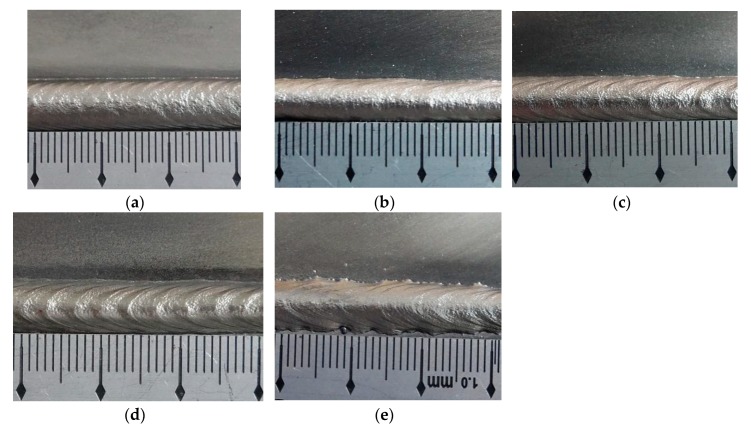
The appearance of the weld bead. (**a**) Specimen CC1, (**b**) specimen CC2, (**c**) specimen CC4, (**d**) specimen CC5, (**e**) specimen CC6.

The appearances of the ripples when employed different twin pulse current changes in percent are shown in Figure 13. As the same as other parts, the weld bead of specimen CC3 was the same as that of specimen I3 in Figure 5c, so Figure 13 included five weld beads. For these experiments, all of weld beads had uniform widths. As for the appearances, the ripples in the weld bead of specimen CC1 was so compacted; the specimen CC2 had compacted but vague ripples. The weld beads of the specimens from CC3 (I3) to CC6 had clear ripples, however, the boundaries of the ripples were clear, and the distances of ripples were large. The overall distances of the ripples were between 2.24 mm and 3.56 mm, and the variation range was 1.32 mm, which was much lower than that in other parts. 

**Figure 14:**
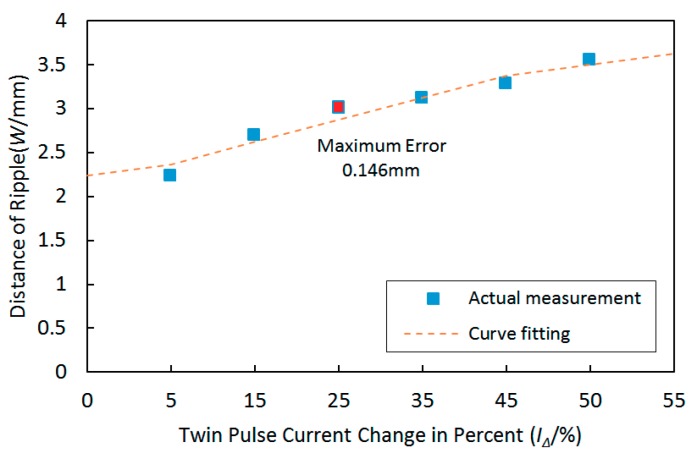
The variation tendency between distances of ripples and twin pulse current change in percent and the curve fitting result.

The variation tendency between twin pulse current change in percent and distances of the ripples is shown in Figure 14. It can be observed that the distances of ripples were increasing with increasing twin pulse current changes in percent, and the variation tendency was also approximately linear.

The corresponding curve fitting equation between twin pulse current changes in percent and distances of the ripples can be described as follows:

y = 0.026x + 2.231(12)

The largest curve fitting error was 0.146 mm, and the corresponding MSE was 0.0092. 

In addition, to present clearly the above experimental analyses, Table 9 shows some more statistical information of the distances of the ripples, which included the maximum value, minimum value, average value, variation range, and standard deviation, collected from the above five arrays of experiments.

In this table, the standard deviation of each array of experiments were provided. Standard deviation can be used to describe the aggregation/dispersion degree of one array of data. In this work, larger value of standard deviation meant that the effect of this operational parameter on the distance of ripples was larger. It can be seen from Table 9 that when employed different welding speeds, the largest value of standard deviation was obtained, which was 1.31, and this situation also corresponded to the largest variation range, which was 3.85 mm. Hence, the results showed that the effect of the welding speed on the distance of ripples was the largest, which was compared to other operational parameters. The following parameter was twin pulse frequency, which corresponded to 0.76 of standard deviation, and the variation range of the distance of ripples were 2.04 mm. The smallest effect was the twin pulse relation, which only corresponded to 0.41 of standard deviation, and the distance of ripples were only 1.16 mm. It meant that the effect of this parameter on the formation of ripples was low.

## 5. Experimental Verification of Curve Fitting

In Section 4, the effects of five main operational parameters on the formation and characteristics of ripples during the DP-GMAW process based on robot operation are provided. Also, corresponding curve fitting equations are supplied for each parameter. In this section, corresponding verifying experiments were conducted to testify the effectiveness of the equations. The special parameter changing was based on the reference experimental conditions of operational parameters. In other word, the operational parameters, apart from the one which was to be verified, used the reference experimental conditions of operational parameters shown in Table 2. Following are the five parameters conditions for experimental verification.

1. The specimen was marked as Y1, set the average current as 85 A;
2. The specimen was marked as Y2, set the welding speed as 35 cm/min;
3. The specimen was marked as Y3, set the twin pulse frequency as 2 Hz;
4. The specimen was marked as Y4, set the twin pulse relation as 35%;
5. The specimen was marked as Y5, set the twin pulse current change in percent as 30%.

**Figure 15:**
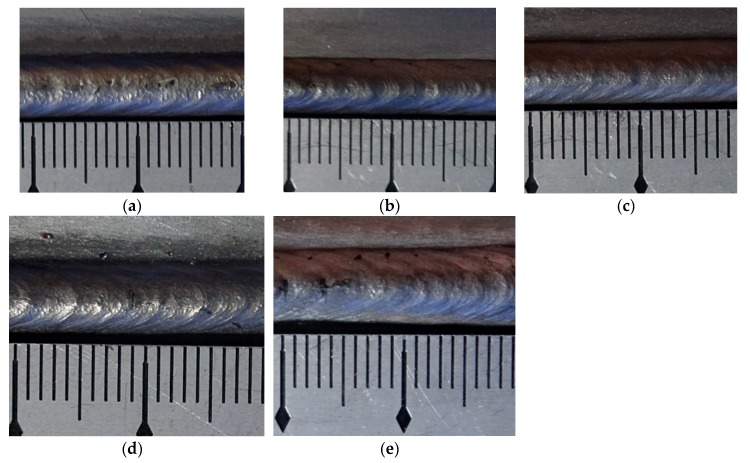
The appearance of the weld bead. (**a**) Specimen Y1, (**b**) specimen Y2, (**c**) specimen Y3, (**d**) specimen Y4, (**e**) specimen Y5.

The experimental results of the appearance of weld beads are shown in Figure 15, while the data collections and analyses are shown in Table 10. 

In this table, all the errors between measurement and curve fitting used the absolute values. According to above experimental results shown in Figure 15 and Table 10, the distances of ripples changed with changing operational parameters. Under the normal variation range of each parameter, corresponding equations can be developed to predict the distances of ripples. In above five testifying experiments, the largest error between measurement and curve fitting equations was 0.315 mm, which was only 9.471% of the measured distance of ripples. The results showed that the equations developed in this work can predict the variation of distances of ripples with small errors under the situation that one operational parameter was changed, and this work can instruct the process improvement and formation control of ripples during the DP-GMAW process. 

## 6. Conclusions and Future Works

This work focused on the effects of some of the main operational parameters on formation and characteristics of ripple, which was a typical output and was considered to be one of the most important quality criterion during the DP-GMAW process. Five main operational parameters, such as average current, welding speed, twin pulse frequency, twin pulse relation, and twin pulse current change in percent, were selected. Reasonable and reliable measurement of distance of ripples were employed in the work to provide convinced and accurate data. Corresponding curve fitting equations between each selected operational parameter and the distances of the ripples were developed according to a series of experiments. During proper variation ranges of five key operational parameters, the variation ranges of the distances of ripples were respectively 1.52 mm, 3.85 mm, 2.04 mm, 1.16 mm, and 1.32 mm, while corresponding standard deviations were respectively 0.51, 1.31, 0.76, 0.41, and 0.42. After a series of analyses and experimental verifications, some conclusions can be drawn as follows:

1. Under the circumstance of using reference experimental conditions of operational parameters, and changed one operational parameter, it can be noticed that the distances of ripples increased with increasing average current, welding speed, and twin pulse current change in percent, and decreased with increasing twin pulse frequency and twin pulse relation.
2. Curve fitting equation can be developed under each main operational parameter within their variation range and distance of ripples to predict the distance. The testifying experiments showed that the effect of each operational parameter on the distance of ripples were approximately linear. In addition, the errors of distances between curve fitting equations and measured values were below 10%.
3. For all of five selected operational parameters, according to the comparisons of variation ranges of the distances of ripples and corresponding standard deviations under five key operational parameters, the most influence on the distance of ripples was the welding speed, and the following was the twin pulse frequency. In addition, the effects of average current on the formation of weld bead was large, and on the distance of ripples was comparatively low. Moreover, the effects of twin pulse current change in percent and twin pulse relation on the distance of ripples were too low. According to the experimental observation and analyses, the ripples were compacted when the distances were between 2 mm and 3 mm, which can obtain satisfied welding process.
4. Though some important conclusions have been obtained in this work, further works are required in the future to study the effects of multi-parameter on the ripples, especially the interactive influences of the welding speed, two current pulses in TPP and TBP, and welding current on the formation and characteristics of ripples.

## Figures and Tables

**Table 1:** Material characteristics of the parent metal and the welding wire.

Materials	C	Si	Mn	Cr	Ni	S	P	N	Mo
304	≤0.08	≤1	≤2	18~20	8~10.5	≤0.03	≤0.03	≤0.1	–
316L	≤0.03	≤1	≤2	16~18	10~14	≤0.03	≤0.045	–	2~3

**Table 2:** Reference experimental conditions of operational parameters in the experiments.

Parameter	Average Current (A)	Welding Speed (cm/min)	Twin Pulse Relation (%)	Twin pulse Current Change in Percent (%)	Twin pulse Frequency (Hz)
Values	80	30	50%	25	1.5

**Table 3:** Experimental plan of the work.

Experimental Index	Average Current (A)	Welding Speed (cm/min)	Twin Pulse Frequency (Hz)	Twin Pulse Relation (%)	Twin Pulse Current Change in Percent (%)
1-average current (I)	**60–110**	30	1.5	50	25
2-welding speed (S)	80	**20–60**	1.5	50	25
3-twin pulse frequency (F)	80	30	**1.5–5**	50	25
4- twin pulse relation (R)	80	30	1.5	**20–80**	25
5-twin pulse current change in percent (CC)	80	30	1.5	50	**5–50**

**Table 4:** Average current and distance of ripples.

Label of Specimen	I1	I2	I3	I4	I5	I6
Average current (A)	60	70	80	90	100	110
Distance of ripples (mm)	2.58	2.46	3.02	3.27	3.4	3.98

**Table 5:** Welding speeds and distance of ripples.

Label of Specimen	S1	S2(I3)	S3	S4	S5
Welding speed (cm/min)	20	30	40	50	60
Distance of ripples (mm)	1.93	3.02	4.2	4.46	5.78

**Table 6:** Twin pulse frequency and distances of ripples.

Label of Specimen	F1 (I3)	F2	F3	F4	F5
Twin pulse frequency (Hz)	1.5	2.5	3.5	4.5	5
Distance of ripples (mm)	3.02	2.32	1.4	1.26	0.98

**Table 7:** Twin pulse relations and distances of the ripples.

Label of Specimen	R1	R2	R3	R4 (I3)	R5	R6	R7
Twin pulse relation (%)	20	30	40	50	60	70	80
Distance of ripples (mm)	3.85	3.89	3.09	3.02	3.09	3.01	2.73

**Table 8:** Twin pulse current change in percent and distances of the ripples.

Label of Specimen	CC1	CC2	CC3 (I3)	CC4	CC5	CC6
Twin pulse current change in percent (%)	5%	15%	25%	35%	45%	50%
Distance of ripples (mm)	2.24	2.7	3.02	3.12	3.29	3.56

**Table 9:** Statistical information of the distances of the ripples collected from the above five arrays of experiments.

Experiment Array	Average Value (mm)	Maximum Value (mm)	Minimum Value (mm)	Variation Range (mm)	Standard Deviation
I1–I6	3.12	3.98	2.46	1.52	0.51
S1–S5	3.88	5.78	1.93	3.85	1.31
F1–F5	1.80	3.02	0.98	2.04	0.76
R1–R7	3.24	3.89	2.73	1.16	0.41
CC1–CC6	2.99	3.56	2.24	1.32	0.42

**Table 10:** Results of experimental verification.

Label of Specimen	Measurement of Distance of Ripples (mm)	Curve Fitting Value of Distance of Ripples (mm)	Error between Measurement and Curve Fitting (mm)	Error of Curve Fitting (%)
Y1	3.186	3.138	0.048	1.507
Y2	3.619	3.407	0.212	5.858
Y3	2.469	2.604	0.135	5.467
Y4	3.248	3.524	0.276	8.498
Y5	3.326	3.011	0.315	9.471
